# Publisher Correction: Maturation of the gut microbiome and risk of asthma in childhood

**DOI:** 10.1038/s41467-018-03150-x

**Published:** 2018-02-13

**Authors:** Jakob Stokholm, Martin J. Blaser, Jonathan Thorsen, Morten A. Rasmussen, Johannes Waage, Rebecca K. Vinding, Ann-Marie M. Schoos, Asja Kunøe, Nadia R. Fink, Bo L. Chawes, Klaus Bønnelykke, Asker D. Brejnrod, Martin S. Mortensen, Waleed Abu Al-Soud, Søren J. Sørensen, Hans Bisgaard

**Affiliations:** 1 COPSAC, Copenhagen Prospective Studies on Asthma in Childhood, Herlev and Gentofte Hospital, University of Copenhagen, Ledreborg Alle 34, 2820 Gentofte, Denmark; 2 Departments of Medicine and Microbiology, and the Human Microbiome Program, New York University Langone Medical Center, 550 First Avenue, New York, NY 10016 USA; 3 Department of Biostatistics, Harvard School of Public Health, 655 Huntington Avenue, Boston, MA 02115 USA; 4 Section of Chemometrics and Analytical Technologies, Department of Food Science, University of Copenhagen, Rolighedsvej 30, 1958 Frederiksberg C, Denmark; 5 Section of Microbiology, Department of Biology, University of Copenhagen, Universitetsparken 15, 2100 Copenhagen, Denmark

Correction to: *Nature Communications* 10.1038/s41467-017-02573-2, published online 10 January 2018

The originally published version of this Article contained an incorrect version of Fig. 3 that was introduced following peer review and inadvertently not corrected during the production process. Both versions contain the same set of abundance data, but the incorrect version has the children’s asthma status erroneously disconnected from the abundance data, thereby producing the non-representative *p* values and graphic presentations. These errors have now been rectified, with the correct version of Fig. 3 replaced in both the PDF and HTML versions of the Article.**Correct version**

 **Incorrect version**

