# Supplementary material for: Maturation of the gut microbiome and risk of asthma in childhood
Source: Nat Commun. 2018 Jan 10;9:141. doi: 10.1038/s41467-017-02573-2 (PMC5762761; doi:10.1038/s41467-017-02573-2)
Supplement: Supplementary file 2 — Supplementary Information [file 41467_2017_2573_MOESM2_ESM.pdf]

SUPPLEMENTARY MATERIALS:

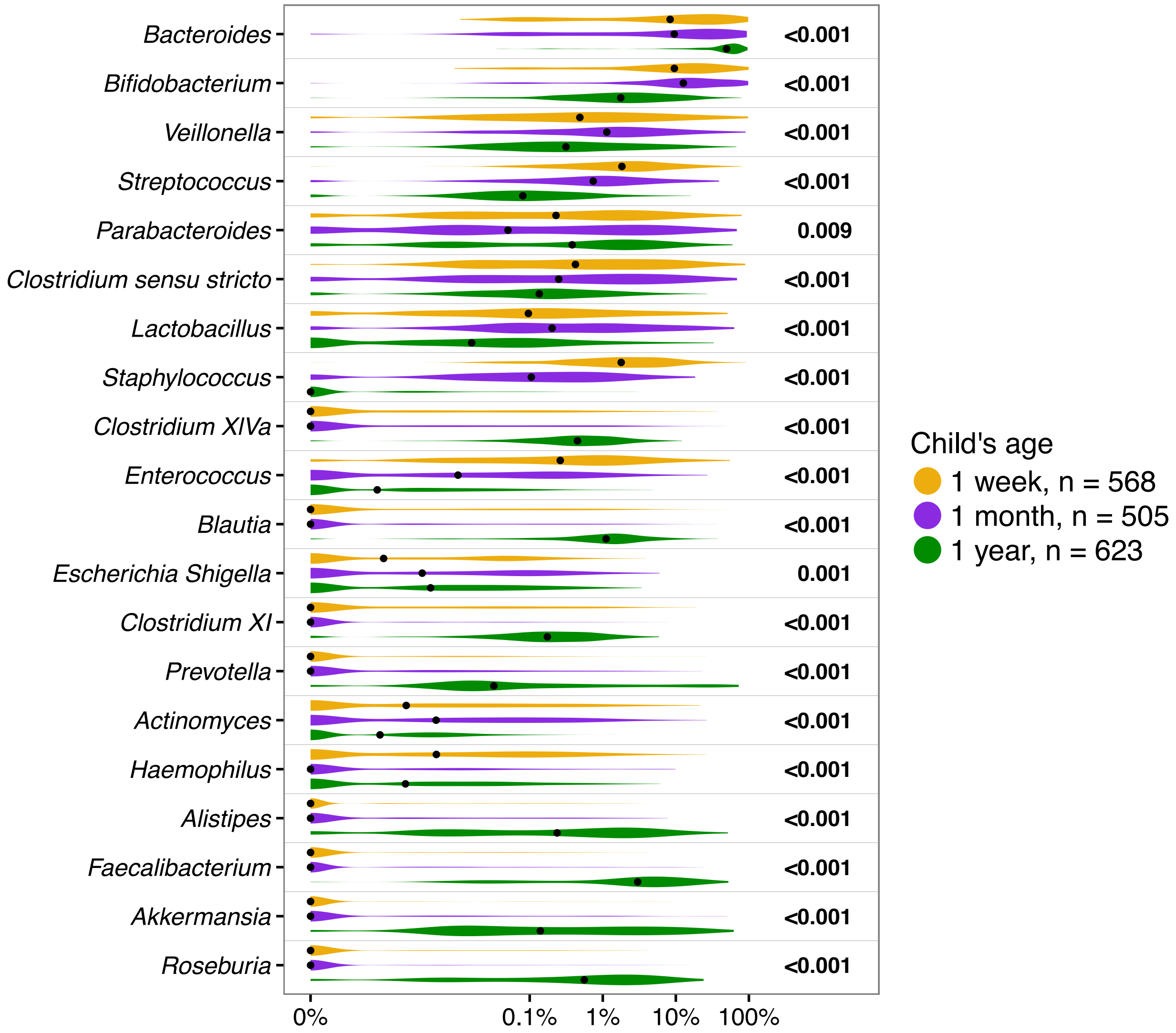

Supplementary figure 1. Relative abundances during the first year of life among the 20 most abundant bacterial genera in all samples, showing the maturation of the microbiota. Comparisons are shown for each genus and the three sampling time-points. P-values correspond to Kruskal-Wallis tests of the relative abundances. FDR limits were calculated for the comparisons: Bonferroni (P<0.0025), Benjamini & Hochberg (P<0.009). A pseudocount (+1e-06) was added to all abundances for the log-scale presentation. The black dots indicate median values.

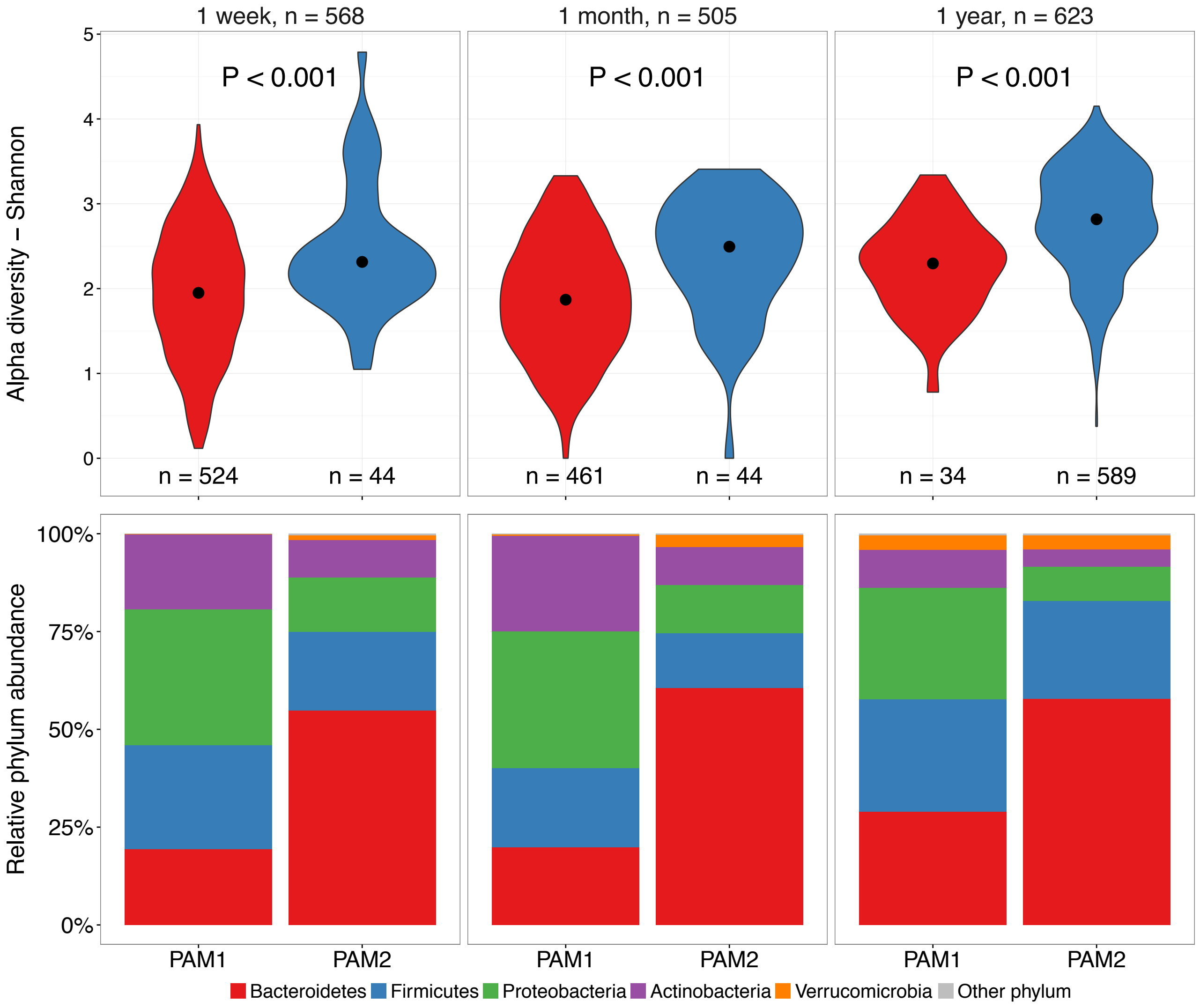

Supplementary figure 2. Shannon diversity and phylum distribution over time, by PAM cluster. Comparisons are shown for Shannon diversity at the three sampling time-points. P-values correspond to Wilcoxon tests. The average relative abundance at different time points are shown at phylum level.

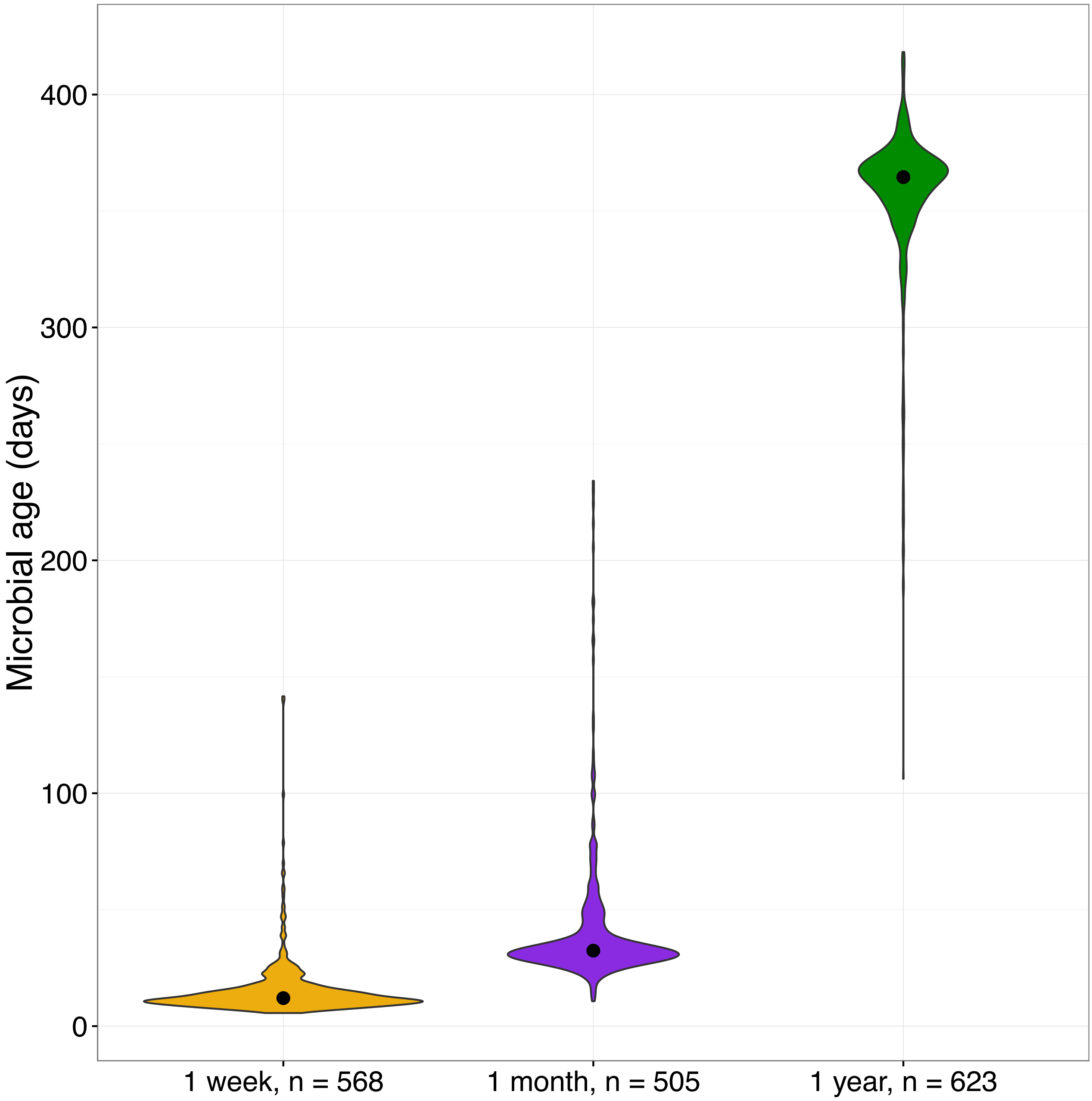

Supplementary figure 3. Microbial age predictions as marker of maturation according to sample time point. The model was trained from the microbial composition on a dataset (all non-asthmatics) with known sample age, and afterward predicted the microbial age for each sample based on the microbial composition alone. Predicted microbial age increased significantly for each time point.

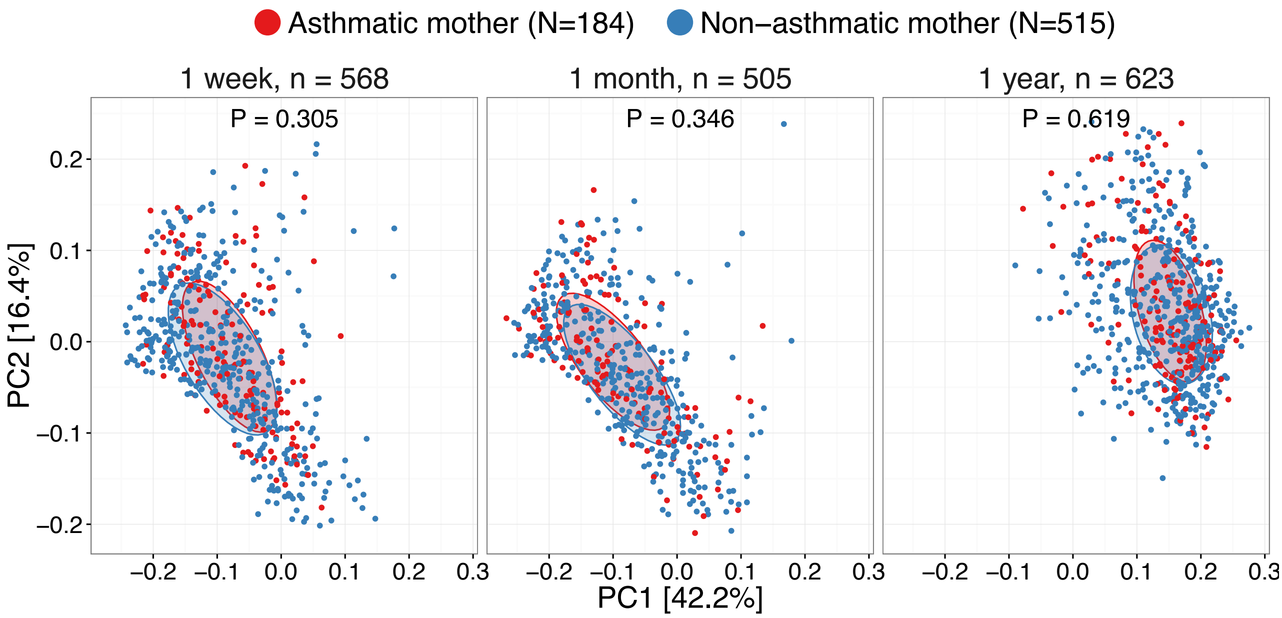

Supplementary figure 4. **β-diversity according to sample time point, as shown by PCoA plots of weighted UniFrac distances.** Microbial compositions were assessed in relation to maternal asthma status. P-values correspond to Adonis PERMANOVA tests. Ellipses demonstrate the mean ±1 SD of children, who has an asthmatic mother (red) (N=184) or a non-asthmatic mother (blue) (N=515).

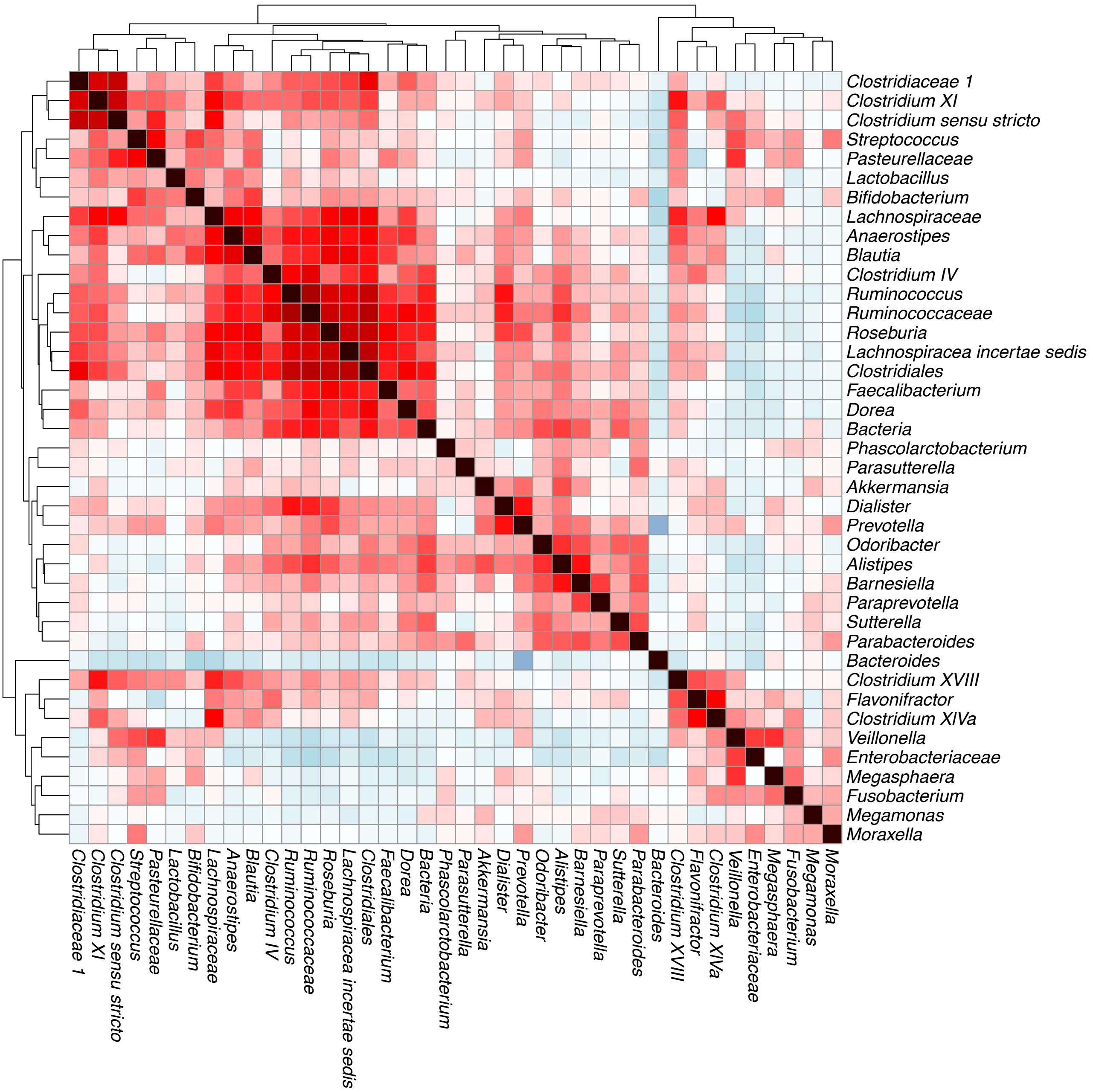

Supplementary figure 5. Heatmap of correlation between genera from 623 samples obtained at 1 year. Spearman correlations, filtered by >10% genus presence and $>10^{-3}$ mean relative abundance. Red and blue colors illustrate positive and negative correlations, repectively.

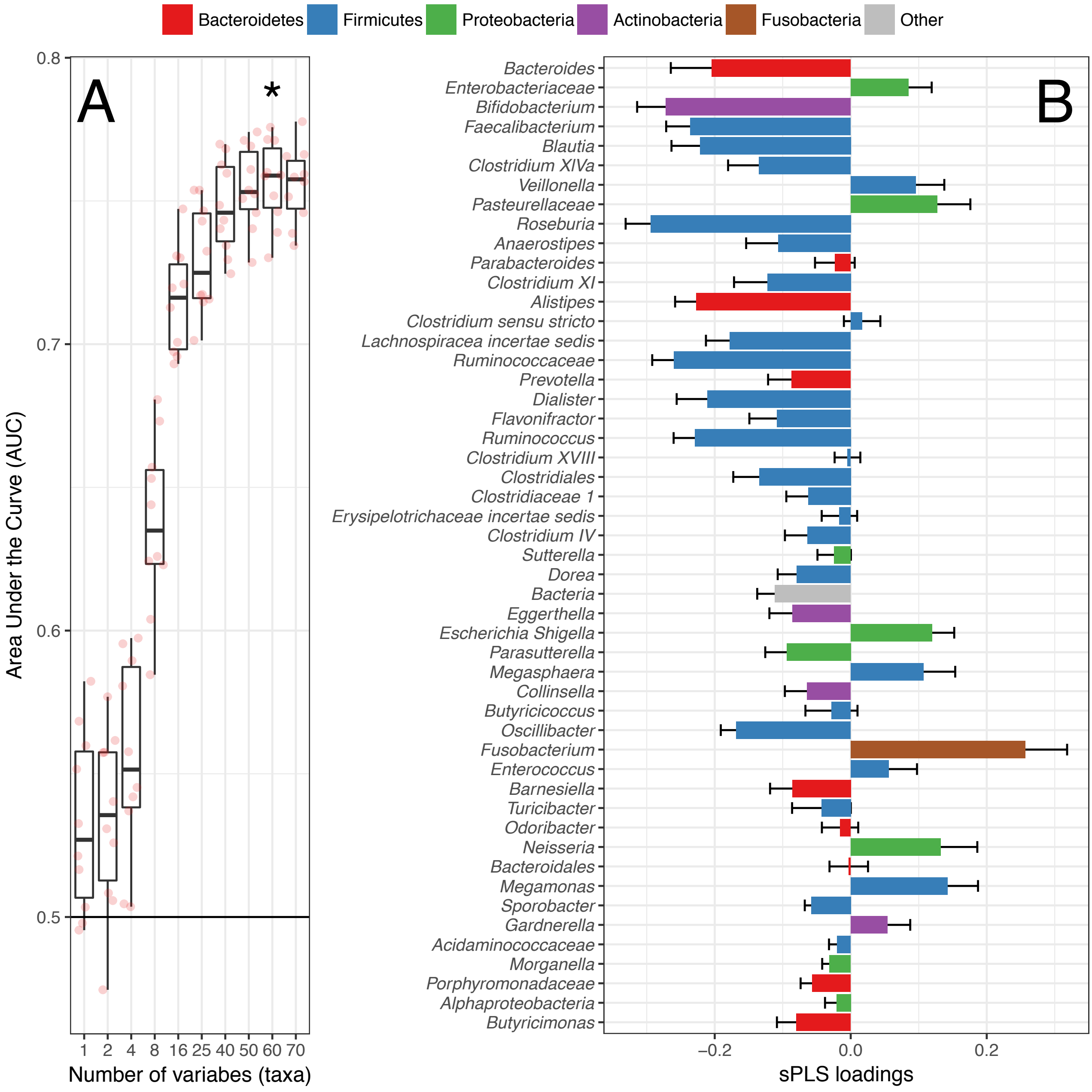

Supplementary figure 6. Sparse Partial Least Squares model predicting asthma at age 5-years in 147 children born by asthmatic mothers. A) AUC results from 10 times repeated 10-fold cross-validation of the models between bacterial genera at age 1-year and asthma at age 5-years (N=147). The asterisk marks the optimal number of variables in the model (n=60) with a median cross-validated AUC of 0.76. B) Loading of each genus in the model with standard deviations across repeats drawn as error bars, ranked by relative abundance and colored by phylum. Taxa with negative loadings were found in lower abundances in asthmatic children and positive loadings were in higher abundances.

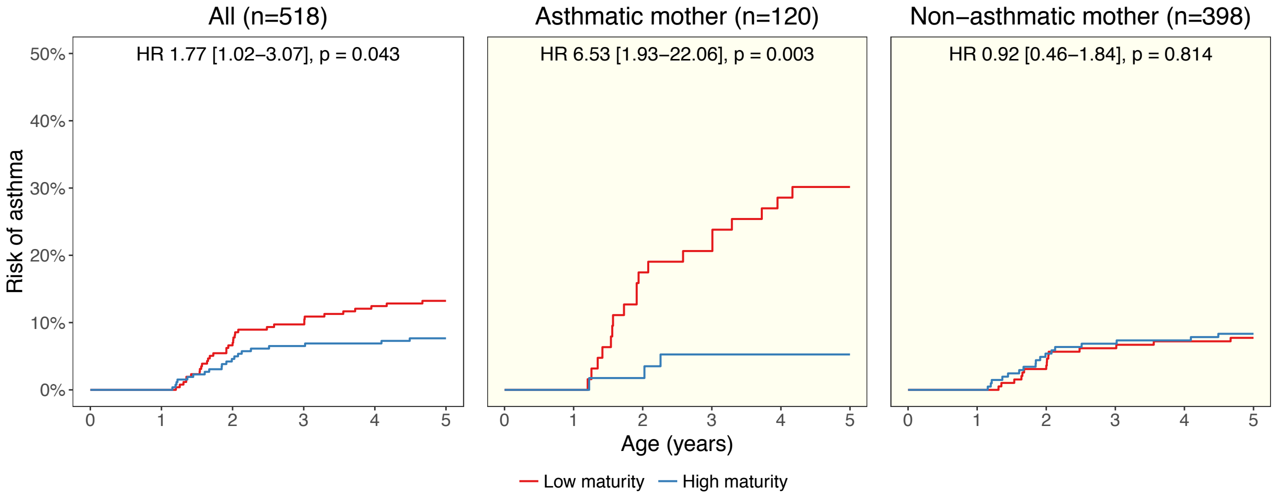

Supplementary figure 7. Kaplan-Meier estimates of cumulative risk of asthma in the first 5 years of life according to microbiota-by-age z-scores (MAZ) at 1 year. Comparisons shown for all children and stratified by maternal asthma, demonstrating the interaction between MAZ and maternal asthma (P=0.006). Associations are quantified by Cox proportional hazards regression, (HR, Hazard Ratio). Curves are colored according to MAZ median-values; low maturity (red) (N=257), high maturity (blue) (N=261). Subsets include: asthmatic mother, low maturity (red) (N=63), high maturity (blue) (N=57); non-asthmatic mother, low maturity (red) (N=194), high maturity (blue) (N=204).
